# Scheimpflug LIDAR for Gas Sensing at Elevated Temperatures

**DOI:** 10.3390/s24237418

**Published:** 2024-11-21

**Authors:** Chet R. Bhatt, Daniel A. Hartzler, Dustin L. McIntyre

**Affiliations:** 1 National Energy Technology Laboratory, 3610 Collins Ferry Road, Morgantown, WV 26505, USA; daniel.hartzler@netl.doe.gov; 2 NETL Support Contractor, 3610 Collins Ferry Road, Morgantown, WV 26505, USA

**Keywords:** Scheimpflug LIDAR, S-LIDAR, Raman-LIDAR, temperature measurement

## Abstract

Localized operating conditions inside boilers, heat recovery steam generators, or other large thermal systems have a huge impact on the efficiency, environmental performance, and lifetime of components. It is extremely difficult to measure species accurately within these systems due to the high temperatures and harsh environments, locally oxidizing or reducing atmospheres, ash, other particulates, and other damaging chemical species. Physical probes quickly suffer damage and are rendered nonfunctional. This work has attempted to adapt the measurement approach based on Scheimpflug light detection and ranging (S-LIDAR) for the remote sensing of gas species inside the high-temperature boiler environment. For a proof-of-concept, the detection of Raman signals of N_2_, O_2_, and CO_2_ and their behavior with increasing temperature have been presented.

## 1. Introduction

Improving energy efficiency in industrial sectors through the implementation of innovative measures and technologies is an integral part of CO_2_ reduction and management. For major industries like metals (e.g., steel and iron), non-metallics (cement, glass, lime), and chemicals, technologies used for the continuous evaluation of chemical species produced or supplied and their physical and chemical properties are crucial. Similarly, the measurement of the temperature inside the production chamber is also equally important to ensure that the process is running at optimized conditions. For chemical sensing and temperature measurement, laser diagnostic techniques are considered the most appropriate in this type of extreme environment, and Raman light detection and ranging (LIDAR) is a promising technique [1,2,3,4]. Raman LIDAR has been extensively used or reported to be useful for remote sensing of atmospheric temperature profiles, water vapor, combustion temperature, CO_2_ leakage at carbon capture and storage sites, gas species concentrations, atmospheric aerosol extinction profiles, etc. [5,6,7,8,9,10,11]. In a conventional LIDAR system, a light pulse is inserted into the target volume, and backscattered laser light is collected to obtain information about the target environment. For a Raman LIDAR system, the backscatter is filtered to remove the elastically scattered light (Rayleigh scatter), while the inelastically scattered light is analyzed to extract information about molecular species within the target volume [3]. In both conventional and Raman LIDAR, typically, a high energy, q-switched, nanosecond pulsed laser is used as a light source, which, along with the required time-resolved detectors and high-power optics, results in a costly system. A comparatively new approach of using a continuous wave (CW) laser instead of a pulsed laser in the LIDAR system operated at a specific optical geometry known as Scheimpflug LIDAR (S-LIDAR) might offer significant cost savings by eliminating the need for a pulsed laser and time-resolved detectors [12,13]. A number of publications have demonstrated S-LIDAR applications in remote sensing [14,15,16,17]. Malmqvist et al. [18] developed a LIDAR system based on the Scheimpflug principle for combustion diagnostics. Atmospheric CO_2_ and NO_2_ sensing are demonstrated by Larsson et al. and Mei et al. using differential absorption LIDAR (DIAL) in the Scheimpflug regime [19,20]. A comparative study of Scheimpflug and pulsed LIDAR techniques for atmospheric aerosol sensing has reported similar temporal and spatial variations observed from the backscattering maps measured during the near horizontal measurements [21]. The authors claimed that the S-LIDAR technique was suitable for the study of planetary boundary layers.

In this work, the Raman S-LIDAR technique has been adapted to evaluate its capability for monitoring gas species inside power generation boilers. Two different Raman scattering regimes were studied: vibrational Raman and rotational Raman. Vibrational Raman scatter relies on the inelastic scatter of light that excites a vibrational mode of a molecule [22,23]. The scattered photons are shifted in energy by an amount equal to the energy of the vibrational modes, allowing identification of molecular species and probing of molecular properties. Like vibrational Raman, pure rotational Raman (referred to as rotational Raman for the purpose of this study) relies on inelastic scattering of light that excites a rotational mode of the molecule without exciting a vibrational mode [24]. Generally, vibrational Raman results in a more significant energy shift of the scattered light (100 s to 1000 s of wavenumber) as compared to rotational Raman (10 s to 100 s of wavenumber), allowing easier identification of Raman bands due to reduced spectral overlap between different molecular species and easier suppression of the Rayleigh scatter. However, rotational Raman scatter, at least for the molecular species studied in this paper, is more intense than the vibrational scatter and has a greater sensitivity to temperature, which is important for thermometry.

## 2. Simulated Boiler Environment

**Figure 1:**
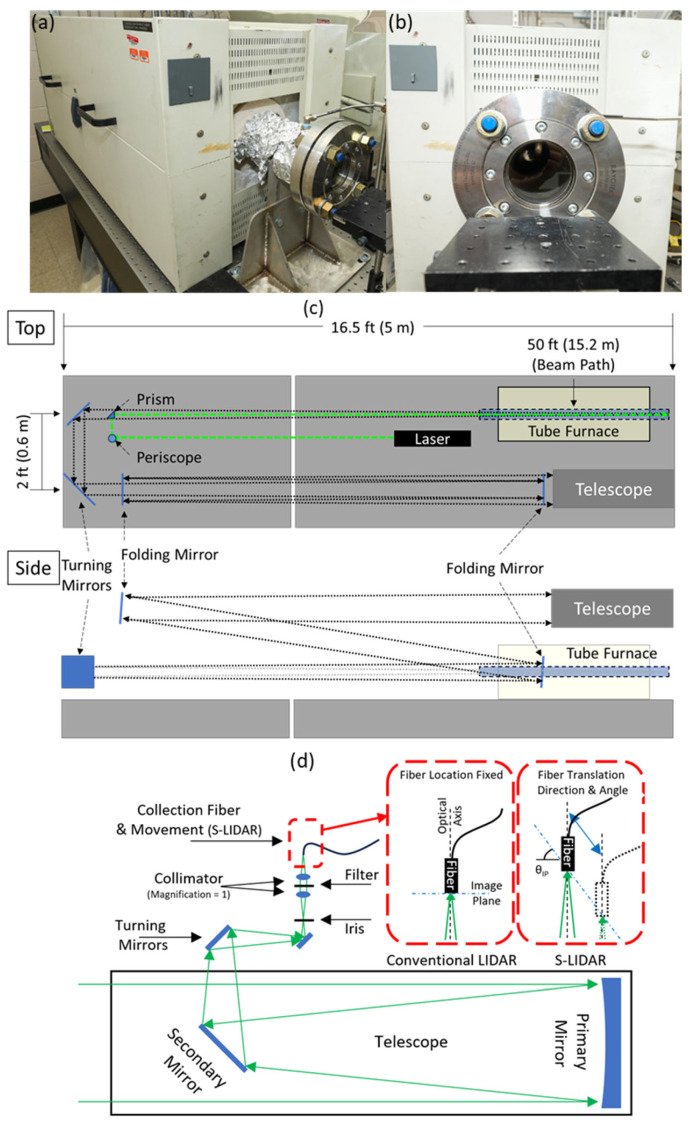
(**a**) Tube Furnace. (**b**) Front window of the Tube Furnace. (**c**) Top view and side view of folded beam path. The full path length is approximately 50 ft/15.2 m from the center of the furnace tube to the front of the telescope. Note: dashed green line is the excitation laser and black dotted lines represent the optical return path to the telescope. (**d**) Telescope collection optics and fiber translation stage. Detail shows the direction of motion of the fiber and the inclination of the image plane (θ_IP_) used for S-LIDAR in this work and also shows the fixed mounting of the fiber used in conventional, pulsed LIDAR. Note: green lines represent the returning Raman scatter from the tube furnace.

A simulated boiler environment was constructed using a tube furnace (Lindberg, HTF55667C Tube Furnace) with optical access via fused silica windows (Rayotek, 3-inch Class 150 Flange, fused silica window) mounted at either end of a 5 ft (1.5 m) long and 3-inch (75 mm) diameter custom stainless steel tube (see Figure 1a,b). The furnace was supplied with variable gas mixtures via a mixing rotameter (Matheson, 7300 Series 2-Tube Mixer (#6 tubes)). Scattered light from the gasses inside the furnace was collected for analysis at several temperature points starting from room temperature. The tube furnace was heated by an electric heating system controlled by a Watlow temperature controller (PM6C1CA-ALAJPWP). Three controllers were used to set the temperature at three different locations to make the temperature uniform across the tube.t Each temperature setting was maintained for >12 h before taking the LIDAR measurements. Gas temperatures were verified by a Type K thermocouple placed approximately 1/3 of the way into the furnace tube. For reference, pulsed LIDAR measurements were also performed at the beginning, and then S-LIDAR measurements were taken. The effect of the temperature on the Raman scattering signals is presented and discussed.

## 3. Pulsed Raman LIDAR Measurements

A Nd: YAG laser (Quantel, Q-smart 850) operated at a wavelength of 532 nm emitting 5 ns, 148 mJ pulses at a 10 Hz repetition rate was used as the light source. The center of the furnace was located approximately 16 m (52 ft) from the telescope (full length of folded beam path, see Figure 1c). The backscattered light was collected by a 250 mm (10-inch), 1 m (39-inch) focal length Newtonian telescope (GSO, GS800) and passed to a Czerny-Turner spectrograph (Andor Technology, Shamrock SR303i-A) via 1000 µm core optical fiber with numerical aperture (NA) of 0.22. Time-resolved detection of the dispersed light was carried out using an intensified charge-coupled device (ICCD) camera (Andor Technology, DH320T-25F-03). Note that a gate width of 7 ns was used for all pulsed measurements, while the gate delay varied depending on the measurement location. To measure the Raman scattering, the tube furnace was filled with Air and nitrogen gas (N_2_) with their varying concentration levels from 0 to 100% (i.e., from approximately 21% O_2_ + 78% N_2_ to 100% N_2_). Measurements were taken at different temperatures ranging from room temperature (approximately 20 °C) to 600 °C. Raman spectra were collected at low (grating 1 = 600 l/mm, 500 nm blaze, 100 µm entrance slit; resolution = 0.55 nm) and high (grating 3 = 2400 l/mm, holographic, 100 µm entrance slit; resolution = 0.12 nm) spectral resolutions settings. The camera gate delay was set such that the volume measured was either centered at the furnace tube mid-point or centered at a point ~6 ft (~2 m) before the front face of the tube (i.e., outside the tube). Data collected inside the tube volume showed sensitivity to the in-tube gas temperature (data follows), while the data collected outside the tube volume showed no sensitivity to the in-tube gas temperature or composition as one would expect for spatially resolved measurements.

**Figure 2:**
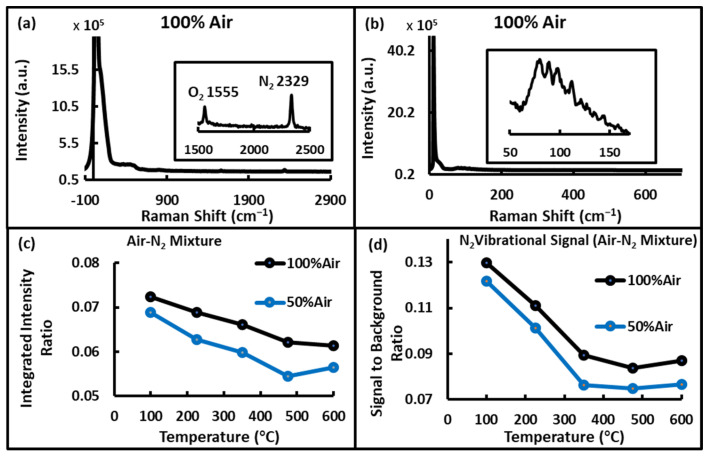
Rotational and vibrational Raman of Air. (**a**) Low-resolution spectrum showing both the (unresolved) rotational bands and O_2_/N_2_ vibrational bands (inset), (**b**) high resolution showing rotational bands, (**c**) variation of the ratio of low to high-frequency rotational Raman band integrated intensities with temperature, and (**d**) variation of N_2_ vibrational signal to background ratio with temperature.

With the lower resolution setting (grating 1), both rotational and vibrational Raman signals could be detected simultaneously; however, the individual rotational bands were not resolved, see Figure 2a. When the highest resolution setting (grating 3) was used, the rotational bands were comparatively resolved, as shown in Figure 2b, though the vibrational bands were outside the observed spectral range. Notably, the individual rotational Raman bands of N2 and O2 are spectrally not well separated (band spacing of 8 cm^−1^ and 12.4 cm^−1^, respectively) [25], which makes it challenging to resolve individual lines, a fact further compounded by the significant spectral overlap between the molecules. However, the measured combined intensity of these lines, using grating 1/low spectral resolution, was approximately 30 times the intensity of the N_2_ vibrational band. In contrast, vibrational Raman lines of N_2_ and O_2_ (band center of 2329 cm^−1^ and 1555 cm^−1^, respectively) have comparatively wider spacing from each other and the Rayleigh line, which makes them feasible for concentration and temperature study even though they are much weaker than the pure rotational Raman lines.

A reduction in the intensity of both the rotational and vibrational signals was observed with the increasing temperature, likely due to changes in gas density with the temperature, suggesting one possible method for determining gas temperature. For example, the signal-to-background ratio of the N_2_ vibrational signal was also found to decrease linearly with an increase in temperature across most of the measured range, as shown in Figure 2d. Additionally, the ratio of the low and high-frequency intensities of the rotational Raman bands was found to decrease almost exponentially with increasing temperatures, as shown in Figure 2c. It must be noted that laser-induced luminescence from the silica windows increased the background, adding noise and degrading the signal. This is particularly disruptive at higher temperatures due to the inherently lower signal.

## 4. Scheimpflug LIDAR (S-LIDAR)

### 4.1. Working Principle

**Figure 3:**
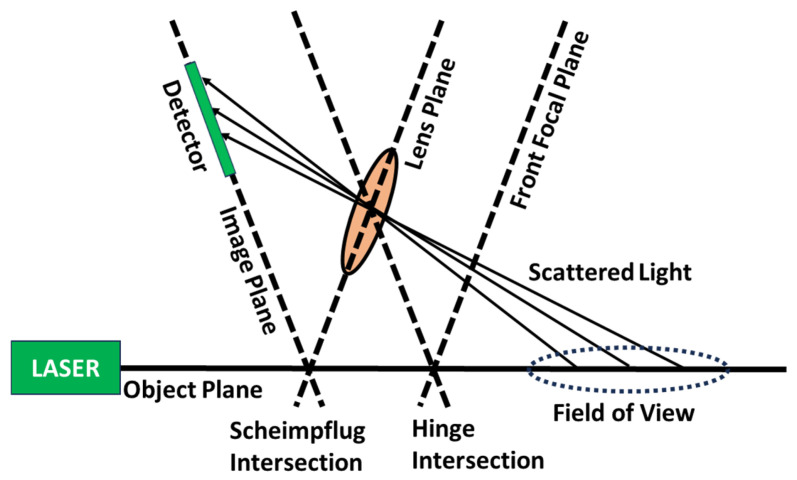
Schematic diagram representing the Scheimpflug condition. Note how points along the laser beam path (the object plane) are mapped to points along the detector.

While conventional LIDAR is based on the time-of-flight measurement principle, which determines distance using the time required for scattered light to return to the instrument after the laser pulse is emitted, the S-LIDAR technique determines distance by mapping the position along the laser beam path onto the position along the image sensor via a special arrangement of the light source, optics, and detector which is known as the Scheimpflug principle. When the image plane is tilted, the plane containing the laser beam (i.e., the object plane) can be brought into focus if the lens plane and image plane intersect the object plane at the same point [16,18]. To obtain the focus, the intersection of the front focal plane of the lens, the object plane, and the image plane displaced to the effective center of the lens must coincide, which is known as the Hinge rule. A schematic diagram representing the Scheimpflug condition is shown in Figure 3.

The S-LIDAR technique significantly reduces the costs and complexity of laser sources and detection equipment as well as the requirement on system components by utilizing low-cost, high-power CW lasers as light sources and highly sensitive, complementary metal oxide semiconductor/charge-coupled device (CMOS/CCD) sensors as detectors as opposed to high-energy pulsed lasers and high-temporal resolution spectrometers required for pulsed LIDAR. The gated detectors required for pulsed LIDAR are also expensive. Infinite depth-of-focus can be achieved by employing large aperture receiving optics. Large aperture receiving optics are especially useful for measurements where the backscattering signals from molecules and particles are weak. In S-LIDAR, the backscattering of a CW laser beam from the volume of interest is sharply imaged onto a detector, and the range-resolved backscattering signals can be retrieved. The ready availability of low-cost, off-the-shelf components has made the S-LIDAR system development a feasible alternative to traditional pulsed LIDAR.

### 4.2. S-LIDAR Experimental Setup

**Figure 4:**
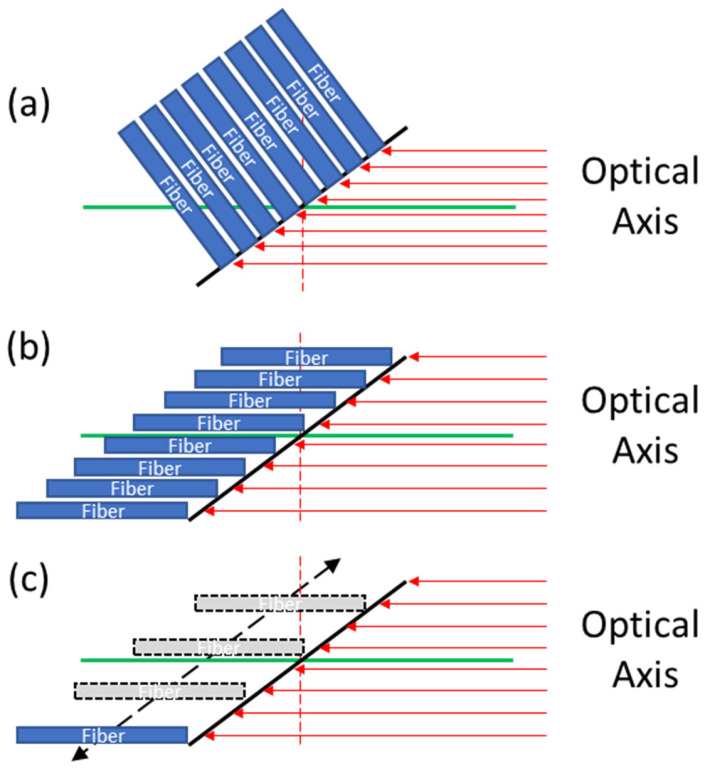
Coupling signal into a fiber/fiber bundle (**a**) using a typical linear bundle, (**b**) using a custom “stepped” bundle, and (**c**) translating a single fiber along the image plane (see Figure 1d). Note: green lines represent the optical axis, red arrows are the incoming signal, solid black lines are the Scheimpflug (tilted) focal plane, dashed black arrow (**c**) is the fiber translation direction, and vertical dashed lines are the non-Scheimpflug (not tilted) focal planes.

The experimental setup used for S-LIDAR measurements was similar to that used in pulsed LIDAR; the major difference is in the light source, the collection system, and excitation laser geometry. For S-LIDAR measurements, a detector such as a CCD can be placed along the image plane, the location and orientation of which can be calculated using Equations (S1) and (S2) (see Supporting Information). However, to perform spectroscopy, the light from the image plane must be coupled into a spectrometer which would require reorienting the image plane to match the focal plane of the spectrometer, specifically the entrance slit. One way this can be done is to use a linear-to-linear fiber optic bundle to collect the signal from the tilted image plane and transfer it to the spectrometer slit. However, the angle of the image plane, together with the light’s travel direction, would result in poor coupling into the fibers (see Figure 4a). This is because the incoming light is incident onto the fiber surface at an angle that far exceeds the acceptance angle/cone of the fiber (as defined by the NA). Possible solutions to this include texturing the fiber ends to refract or scatter light into the acceptance cone of the fibers; frosting the glass is perhaps the simplest (though lossy) method. Alternatively, producing a “stepped” linear fiber bundle (Figure 4b) would permit the focused light to be directly coupled into the fiber.

For proof-of-concept testing, a single 1000 µm core fiber was mounted on a miniature ½”/12.7 mm translation stage (ThorLabs, DT12) with its face perpendicular to the optical axis and moved along the image plane to collect data one point at a time as opposed to collecting the full image plane at once (Figure 4c). Each point along the laser within the tube maps to a unique point along the image plane, given by Equation (S4), permitting a distance-resolved distribution of temperature and chemical species to be determined. While this expression is not a linear function of distance from the telescope, it is approximately linear across the length of the tube used in this experiment (see Figure S4).

The use of a linear fiber bundle would permit simultaneous measurement of multiple points along the excitation laser path. Since each fiber core collects light from a specific point, an imaging spectrograph would be able to measure the spectrum of each core independently.

**Figure 5:**
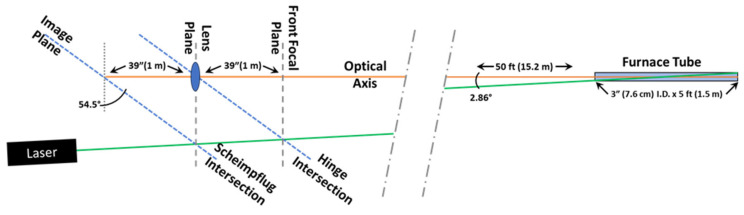
Simplified representation of the optical system demonstrating satisfaction of the Scheimpflug condition. In this representation, all folding mirrors (see Figure 1c) have been removed, and the telescope has been replaced by a 39-inch/1 m focal length lens. The figure is approximately to scale. Note: green line is the excitation laser, and the angled dash-dot lines indicate where the figure has been shortened.

For measurements, a CW green laser (Opto Engine, MGL-N-53A-5W) operating at a wavelength of 532 nm was used. The beam entered the 3-inch (75 mm) I.D. × 5 ft (1.5 m) tube at an angle of approximately 2.86° relative to the tube axis, intersecting the optical axis at roughly the center of the tube (defined as the center of the object plane). With the object plane located 50 ft (1.52 m) and inclined at an angle of 2.86° relative to the optic axis, for a 39-inch (1 m) focal length telescope, the angle of the image/detector plane was determined to be 54.5° from vertical using Equation (S2) (see Supporting Information). This is demonstrated graphically in Figure 5, using a simplified, to-scale (approximate) representation of the optical system, showing the Scheimpflug condition is satisfied for the angles and distances involved.

For the specific experimental S-LIDAR setup used in this study (as described above), all points along the full 5 ft (1.52 m) length of the furnace tube fall within a 9.5 mm length along the image plane (as calculated by Equation (S3)), allowing the 12.7 mm translation stage to access all points within the furnace and some distance outside the tube. This fact was used to both align the system by placing targets with known spectral properties (e.g., a fluorescent dye or Raman standard such as acetonitrile or sulfur) at either end of the tube and to verify the ability of the system to spatially resolve measurements by collecting spectra from the tube center (data below) and from just outside the tube showing room temperature air outside the tube regardless of the temperature/gas mixture inside. This data is provided in a later section (Section 4.3.3).

Additionally, the spatial resolution of this S-LIDAR setup is estimated to be approximately 11″ (280 mm) at the tube center and varies from 10″ to 12.5″ (253–318 mm) across the length of the tube as calculated from Equation (S5), meaning the system collects a signal from a region that extends some distance (±5″/127 mm to ± 6″/152 mm in this case) to either side of the measurement point. While this is primarily due to the finite size of the collection fiber (see Equation (S5)), the resolution also depends on the distance to the measurement point, with the resolution worsening the larger the distance from the telescope. Note that although the expression for spatial resolution is not a linear function of distance, it is approximately linear across the length of the tube used in this experiment (see Figure S4).

### 4.3. Scheimpflug Lidar Measurements

For the measurements, two different gas combinations, Air-N_2_ and CO_2_-N_2_, were supplied into the furnace tube separately (note: the air was dry and assumed 78% N_2_/21% O_2_/1% other (ignored)). The concentration of the constituent gases was varied to make five different mixtures for each combination, as presented in Table 1 (actual concentrations of N_2_ and O_2_ are provided for the Air-N_2_ mixtures). A CW laser (5 W, 532 nm) was incident into the furnace tube to interact with the gas inside the tube. For each gas mixture, measurements were taken at temperatures 20 °C (room temperature), 100 °C, 225 °C, 350 °C, 475 °C, and 600 °C separately. The spectrometer gate width was kept at 100 ms. Like in pulsed LIDAR, measurements were taken using low (grating 1; 600 L/mm) and high (grating 3; 2400 L/mm) resolution settings. The low-resolution grating gives spectra in the Raman shift range of 0–3655 cm^−1^, while with high-resolution grating, spectra were recorded in the Raman shift range of 0–685 cm^−1^. The optical fiber mounted on a translation stage was moved to collect data from the middle of the furnace tube and outside the front window of the tube.

#### 4.3.1. Scheimpflug LIDAR Signals Detection

**Figure 6:**
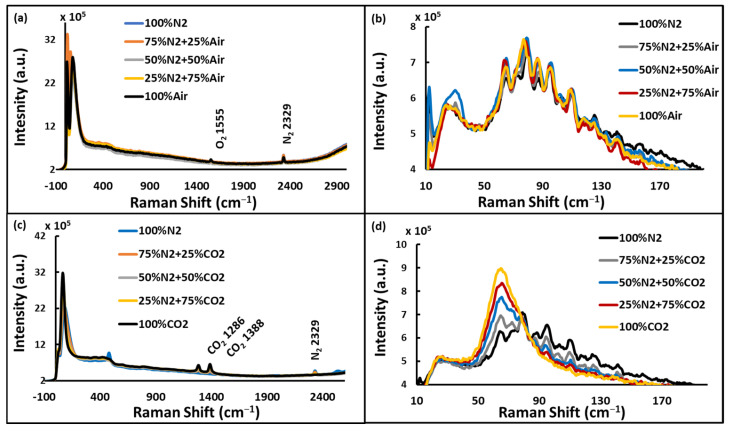
Room temperature rotational and vibrational bands of N_2_, O_2_, and CO_2_ observed for different gas mixtures. (**a**) N_2_ and O_2_ vibrational signals from N_2_-Air mixture with 600 L/mm resolution, (**b**) N_2_ rotational band from N_2_-Air mixture with 2400 L/mm resolution, (**c**) N_2_ and CO_2_ vibrational signals from N_2_-CO_2_ mixture, and (**d**) rotational signals from N_2_-CO_2_ mixture (note the lack of resolved bands in the 100% CO_2_ spectrum).

Raman spectra of the varying concentrations of Air, N_2_, and CO_2_ in the tube furnace at room temperature (20 °C) are shown in Figure 6. Both the rotational and vibrational Raman signals of the gases were observed with lower resolution grating, as shown in Figure 6a,c. However, the rotational bands were not resolved with this grating. Therefore, the higher resolution grating was used to obtain rotational bands (Figure 6b,d), although they were not completely resolved. It should be noted that these bands sit atop a pedestal extending from less than 50 cm^−1^ to over 500 cm^−1^, consisting of overlapping bands from the fused silica window of the furnace tube [26]. With the grating of 600 L/mm, the oxygen band was observed with its maxima at 1555 cm^−1^, N_2_ band at 2329 cm^−1^, and CO_2_ bands at 1286 cm^−1^ and 1388 cm^−1^ in the spectra. As expected, vibrational band strength was observed to increase with increasing concentration. Partially resolved rotational bands were observed in the range 60–180 cm^−1^ when the gas mixture contained Air or N_2_. However, for CO_2_, the rotational bands were unresolved even at the highest resolution setting (see Figure 6d). This is due to the much smaller rotational band spacing for CO_2_ (3.1 cm^−1^) as compared to N_2_ (8.0 cm^−1^) and O_2_ (11.4 cm^−1^) [25].

As previously stated, in this experiment, only one optical fiber was used to collect the data from the center of the tube. However, data can be collected simultaneously from multiple locations using multiple fibers. This shows that S-LIDAR might be useful for gas composition detection within the boiler environment, and with proper calibration, quantitation of detected gas may be performed.

#### 4.3.2. Scheimpflug LIDAR Measurements at Elevated Temperatures

**Figure 7:**
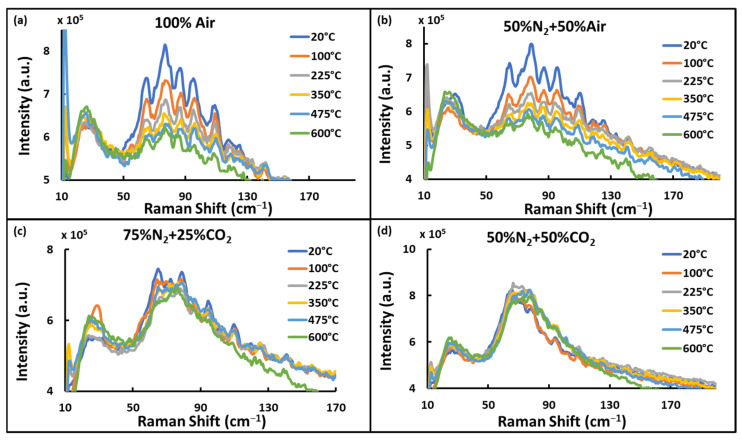
Rotational Raman spectra obtained at the temperature range of 20–600 °C. (**a**) Spectra from 100% Air, (**b**) spectra from a mixture of 50% N_2_ and 50% Air, (**c**) spectra from a mixture of 75% N_2_ and 25% Air, and (**d**) spectra from a mixture of 50% N_2_ and 50% CO_2_.

To evaluate the S-LIDAR measurements at elevated temperatures, the tube furnace was heated from room temperature (20 °C) to 100 °C, 225 °C, 350 °C, 475 °C, and 600 °C sequentially for each gas mixture inside the tube. Both high and low-resolution spectra were recorded from each gas mixture at each temperature. Rotational signals at different temperatures for some selected gas mixtures are shown in Figure 7. For Air and N_2_ mixtures, rotational signal intensity decreased with the increasing temperature, as displayed in Figure 7a,b. For CO_2_-N_2_ mixtures, rotational spectra were observed as shown in Figure 7c,d, where higher CO_2_ content caused poor spectral resolution, though a shift in the maximum toward higher wavenumber is observed as expected.

**Figure 8:**
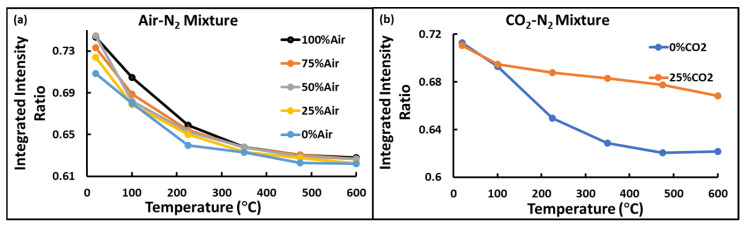
Variation of the ratio of low and high-frequency rotational Raman band integrated intensities with increasing temperature (**a**) in Air-N_2_ mixture, and (**b**) in CO_2_ and N_2_ mixture.

To understand the behavior of rotational signals with increasing temperatures, the ratio of low to high-frequency integrated band intensities (background subtracted) was plotted against temperature. For Air-N_2_ mixtures, like in pulsed LIDAR, the integrated intensity ratio decreased almost exponentially with increasing temperature, as shown in Figure 8a. For CO_2_-N_2_ mixtures, as mentioned above, high CO_2_ content caused poor resolution. Therefore, rotational bands of low CO_2_ content mixtures were considered. The integrated intensity ratio decreased with increasing temperature, as shown in Figure 8b.

**Figure 9:**
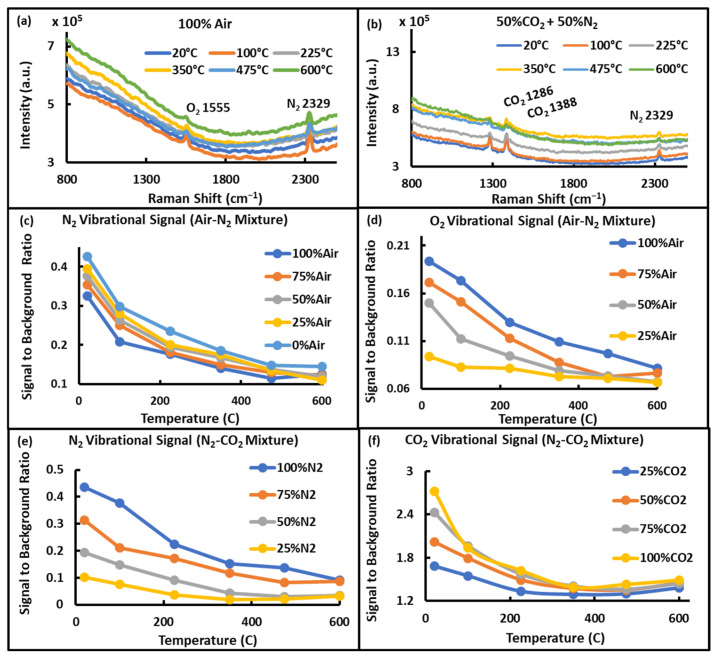
(**a**) Spectra showing O_2_ and N_2_ vibrational signals obtained from 100% Air. (**b**) Spectra showing CO_2_ and N_2_ vibrational signals obtained from the mixture of 50% CO_2_ and 50% N_2_. (**c**) Variation of N_2_ vibrational signal to background ratio with increasing temperature in the Air-N_2_ mixture. (**d**) Variation of O_2_ vibrational signal to background ratio with increasing temperature in the Air-N_2_ mixture. (**e**) Variation of N_2_ vibrational signal to background ratio with increasing temperature in the N_2_-CO_2_ mixture. (**f**) Variation of CO_2_ vibrational signal to background ratio with increasing temperature in the N_2_-CO_2_ mixture.

Similar to the results observed with pulsed LIDAR measurements, the intensity of vibrational signals was also observed to decrease with increasing temperature, as shown in Figure 9. The decreasing intensity of O_2_ and N_2_ signals from 100% Air is displayed in Figure 9a, and that of CO_2_ and N_2_ from the mixture having equal content of CO_2_ and N_2_ is shown in Figure 9b. Signal-to-background ratios of these vibrational bands of N_2_, O_2_, and CO_2_ were plotted against the temperature to study the effect of increasing temperature, using the 2329 cm^−1^ and 1555 cm^−1^ vibrational Raman bands of N_2_ and O_2,_ respectively, and the summed intensity of the 1286 cm^−1^ and 1388 cm^−1^ CO_2_ bands. The decreasing trends of the intensity of bands of N_2_, O_2_, and CO_2_ were almost exponential with the increasing temperature, as demonstrated in Figure 9c–f. These decreasing trends were similar to those found with pulsed LIDAR measurements, which shows that the S-LIDAR method can be used instead of pulsed LIDAR for gas species measurements in atmospheric as well as high-temperature environments.

#### 4.3.3. Spatial Resolution

**Figure 10:**
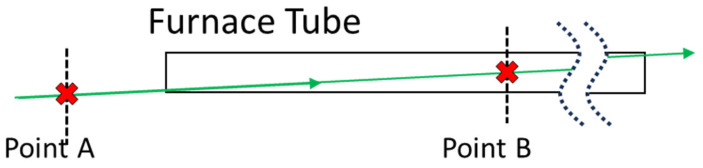
Locations along the furnace tube where S-LIDAR measurements were made. Point A lies just outside the tube’s front face, while Point B lies at the tube center. Note: the green line is the excitation laser.

Spatial resolution was demonstrated by taking data at two points, one inside and one outside of the furnace tube (Figure 10). The “inside” point lies approximately at the tube center (see “Point B” in Figure 10) and is where all temperature/gas species measurements were performed, while the “outside” point lies just outside the front face of the tube. These points were selected as they minimized the spectral interference from the tube window, both from fluorescence and silica Raman, and each point could be accessed at will, without the need for realignment of the optical system, by means of the translation stage used to move the collection fiber along the image plane (see Figure 1d and Figure 4c).

**Figure 11:**
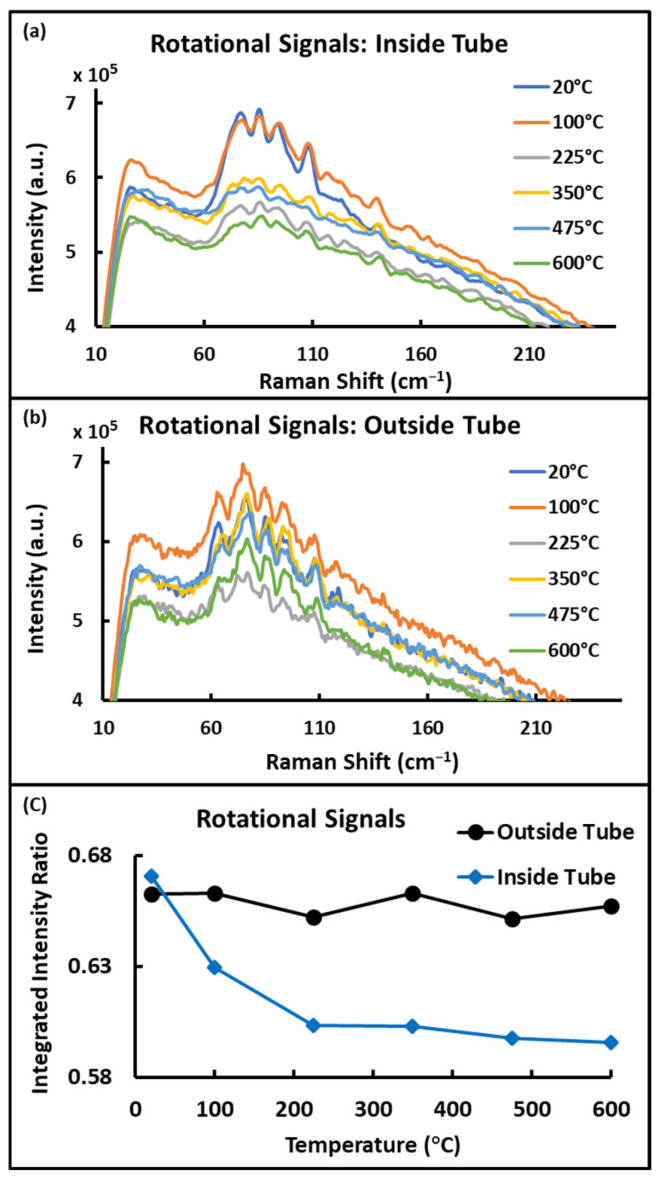
(**a**) Rotational Raman spectra measured at the tube center/Point B. (**b**) Spectra collected from outside the tube/Point A showing no sensitivity to the in-tube temperature. (**c**) Variation of the ratio of low and high-frequency rotational Raman band integrated intensities with increasing temperature for points at the tube center (blue) and just outside the front face of the tube (black). This result demonstrates the ability of the described system to spatially resolve gas properties such as temperature.

Data collected at Point B (Figure 10) showed sensitivity to temperature, observable in both the intensity ratio of low and high-frequency rotational Raman band (described above) and in the signal intensity reduction due to rarefaction of the gas at higher temperatures (also described above), while data collected at Point A showed no sensitivity to the in-tube temperature (see Figure 11). The ability to select these two points, separated by 3 ft (1 m), clearly demonstrates the ability of this method to provide useful spatial resolution. Such a system is even able to provide simultaneous measurements of multiple points along the excitation laser’s path using a method described in Figure 4b. Note that the data shown in Figure 11 was collected concurrently, meaning that once the gas temperature stabilized, data was collected at both Point A and Point B (Figure 10) before moving on to the next temperature set point.

#### 4.3.4. Peak Shifting with Temperature

**Figure 12:**
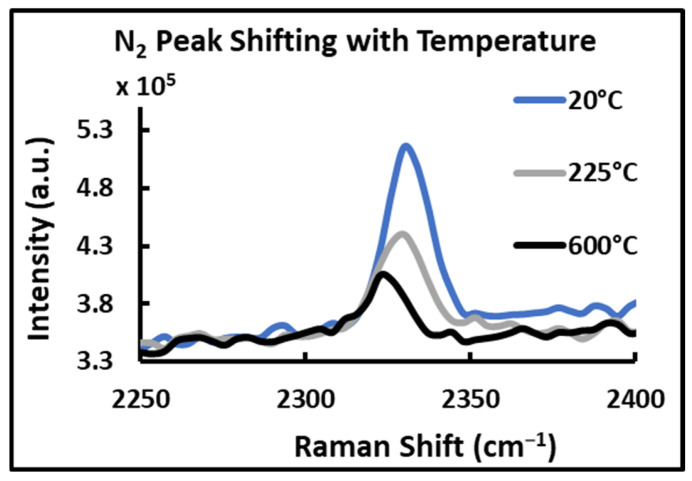
N_2_ vibrational signal peak shifting with increasing temperature.

Changes in conditions like pressure and temperature can stimulate the attractive and repulsive forces between the molecules, which can result in the Raman peak position shifting [27,28,29,30]. When temperature increases, the bond length increases, and hence, the energy of the vibrational mode may decrease. Similarly, a decrease in temperature causes bond length shortening, and hence, vibrational mode energy increases. Though the pressure was not taken into account in this study, the N_2_ peak position was evaluated to determine if its shifting with the change in temperature can be noticed. When spectra taken from N_2_ gas at different temperatures were stacked together, some shifting of the N_2_ vibrational signal was observed, as shown in Figure 12; however, as the vibrational signals were recorded at low-resolution settings only, exact shifting could not be presented quantitatively. This may be a possibility for future investigation.

## 5. Conclusions

A S-LIDAR system has been developed to demonstrate the proof-of-concept for use in gas sensing in high-temperature environments. Measurements were taken from two different gas mixtures, Air-N_2_ and CO_2_-N_2_, which were filled in the tube furnace in temperatures ranging from room temperature (20 °C) to 600 °C. Both rotational and vibrational signals were evaluated at increasing temperatures. While the ratio of low and high-frequency rotational Raman band integrated intensities was considered to determine the effect of temperature on rotational signals, the background ratio was evaluated on vibrational signals. Both were found to decrease almost exponentially with increasing temperatures. Similar results were observed when measurements were taken from the Air-N_2_ mixture with pulsed LIDAR. Hence, these results show that Raman signals of gases can be detected to claim their presence in the target environment with the S-LIDAR system with adequate detection resolution. Moreover, proper evaluation of the change in the Raman signal pattern may be useful to obtain indicative information about changes in the environment. For example, an increase in CO_2_ content can be known by examining the CO_2_ signal strength itself or the rotational spectrum of N_2_, which might be useful to take further action for the efficient continuation of power plant boilers and for the purpose of carbon management.

## Figures and Tables

**Table 1:** Gas compositions were used for the measurements.

Air-N_2_ Mixtures			CO_2_-N_2_ Mixtures	
Air%	N_2_%	Actual N_2_%/O_2_%	CO_2_%	N_2_%
100	0	78/21	100	0
75	25	83.5/15.8	75	25
50	50	89.0/10.5	50	50
25	75	94.5/5.2	25	75
0	100	100/0	0	100

## Data Availability

Data are contained within the article.

## Supplementary Materials

The following supporting information can be downloaded at https://www.mdpi.com/article/10.3390/s24237418/s1.

## Disclaimer

This project was funded by the United States Department of Energy, National Energy Technology Laboratory, in part through a site support contract. Neither the United States Government nor any agency thereof, nor any of their employees, nor the support contractor, nor any of their employees, makes any warranty, express or implied, or assumes any legal liability or responsibility for the accuracy, completeness, or usefulness of any information, apparatus, product, or process disclosed, or represents that its use would not infringe privately owned rights. Reference herein to any specific commercial product, process, or service by trade name, trademark, manufacturer, or otherwise does not necessarily constitute or imply its endorsement, recommendation, or favoring by the United States Government or any agency thereof. The views and opinions of authors expressed herein do not necessarily state or reflect those of the United States Government or any agency thereof.
