# Supplementary material for: Scheimpflug LIDAR for Gas Sensing at Elevated Temperatures
Source: Sensors (Basel). 2024 Nov 21;24(23):7418. doi: 10.3390/s24237418 (PMC11644416; doi:10.3390/s24237418)
Supplement: Supplementary file 1 [file sensors-24-07418-s001.zip › sensors-3211083-supplementary.pdf]

Scheimpflug LIDAR for Gas Sensing at Elevated Temperatures - SUPPORTING INFORMATION

Chet R Bhatt[1,2]*; Daniel A Hartzler*[1,2]*; Dustin L McIntyre*[1]

[1]*National Energy Technology Laboratory, 3610 Collins Ferry Road, Morgantown, WV 26505, USA*

[2]*NETL Support Contractor, 3610 Collins Ferry Road, Morgantown, WV 26505, USA*

Section 1: Geometric relation between excitation laser and image plane.

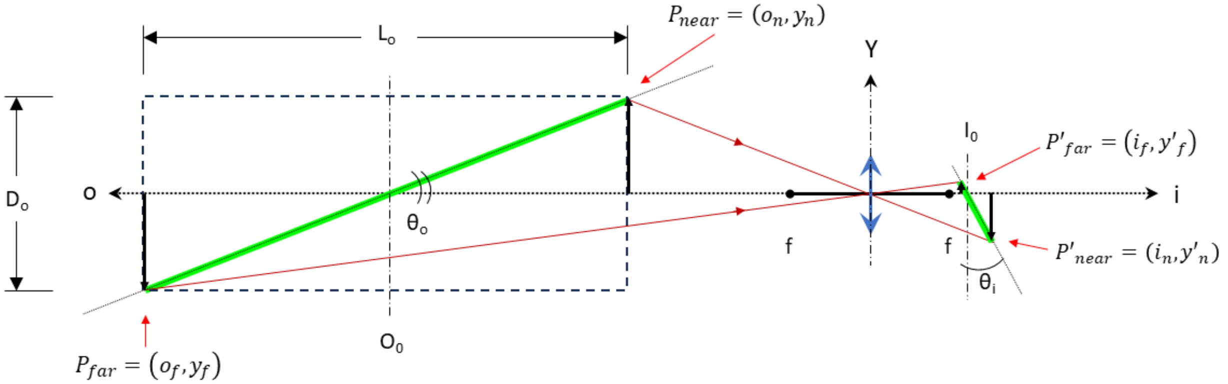

Figure S1 *Geometry of S-LIDAR system. Note that the figure follows the Gaussian sign convention with the object ('o') and image ('i') distances, originating at the focusing element (blue double arrow) with +o pointing to the left and +i to the right while +Y points up.*

The furnace tube, length 'L_o' and diameter 'D_o', is located a distance 'O_0' (center of tube) from the focusing element (focal length 'f') while the laser passes through the tube at an angle of 'θ_o' with respect to the optical axis, passing through points 'P_{near}' and 'P_{far}' as well as the center of the furnace at 'O_0' (see **Figure S1**).

The points P_{near} and P_{far} and the angle θ_o are set by the fixed system geometry by the following:

Object / Laser plane parameters:

$$\boldsymbol{P_{near}} = (o_n, y_n) = \left(\boldsymbol{O_0} - \frac{\boldsymbol{L_o}}{\boldsymbol{2}}, \frac{\boldsymbol{D_o}}{\boldsymbol{2}}\right)$$

$$\boldsymbol{P_{far}} = (o_f, y_f) = \left(\boldsymbol{O_0} + \frac{\boldsymbol{L_o}}{\boldsymbol{2}}, -\frac{\boldsymbol{D_o}}{\boldsymbol{2}}\right)$$

$$\tan \boldsymbol{\theta_o} = {}^{\boldsymbol{D_o}}/_{\boldsymbol{L_o}}$$

Points $P_{near,far}$ can be mapped onto points along the image plane, $P'_{near,far}$ (see **Figure S1**), by means of the lens equation and system magnification, 'M':

$$P'_{near,far} = \left(i_{n,f}, y'_{n,f}\right)$$

Where: $i_{n,f} = \frac{o_{n,f}*f}{(o_{n,f}-f)}$, $y'_{n,f} = M_{n,f} * y_{n,f},\ and\ M_{n,f} = \frac{-i_{n,f}}{o_{n,f}} = \frac{-f}{(o_{n,f}-f)}$

Substituting in the expressions for $o_{n,f}$ and $y_{n,f}$ into P'$_{near,far}$ yields expressions for the endpoints of the image plane:

$$\boldsymbol{P'_{near}} = (i_n, y'_n) = \left(\frac{o_n * f}{(o_n - f)}, \frac{y_n * f}{(o_n - f)}\right) = \left(\frac{\left(\boldsymbol{O_0} - \frac{\boldsymbol{L_o}}{2}\right)\boldsymbol{f}}{\left(\left(\boldsymbol{O_0} - \frac{\boldsymbol{L_o}}{2}\right) - \boldsymbol{f}\right)}, \frac{-\frac{\boldsymbol{D_o}}{2}\boldsymbol{f}}{\left(\left(\boldsymbol{O_0} - \frac{\boldsymbol{L_o}}{2}\right) - \boldsymbol{f}\right)}\right)$$

$$\boldsymbol{P'_{far}} = (i_f, y'_f) = \left(\frac{o_f * f}{(o_f - f)}, \frac{y_f * f}{(o_f - f)}\right) = \left(\frac{\left(\boldsymbol{O_0} + \frac{\boldsymbol{L_o}}{2}\right) * \boldsymbol{f}}{\left(\left(\boldsymbol{O_0} + \frac{\boldsymbol{L_o}}{2}\right) - \boldsymbol{f}\right)}, \frac{-\frac{\boldsymbol{D_o}}{2} * \boldsymbol{f}}{\left(\left(\boldsymbol{O_0} + \frac{\boldsymbol{L_o}}{2}\right) - \boldsymbol{f}\right)}\right)$$

From which expressions for the angle of the image plane with respect to the vertical axis, 'θ_i', intersection with the horizontal axis, 'I_0', and image plane length 'L_I' can be calculated:

Image Plane Parameters:

$$\boldsymbol{I_0} = \frac{\boldsymbol{O_0} * \boldsymbol{f}}{(\boldsymbol{I_0} - \boldsymbol{f})}$$

Equation S1 *Intersection of image plane with optical axis measured from the focusing element (telescope's lens/mirror), see* ***Figure S1****. This distance roughly defines the location of the detector with respect to the focusing element.*

$$\tan \boldsymbol{\theta_i} = -\frac{\boldsymbol{L_o}}{\boldsymbol{D_o}} \frac{\boldsymbol{f}}{(\boldsymbol{O_0} - \boldsymbol{f})}$$

$$\tan \boldsymbol{\theta_i} = -\frac{\boldsymbol{f}}{\tan \boldsymbol{\theta_o} (\boldsymbol{O_0} - \boldsymbol{f})}$$

Equation S2 *Angle of the image plane with respect to the vertical axis (i.e., the axis perpendicular to the optical axis). This angle defines the "off vertical tilt" of the detector (see* ***Figure S1****).*

$$\boldsymbol{L_I} = \frac{\boldsymbol{f}\sqrt{(\boldsymbol{L_o f})^2 + \boldsymbol{D_o}^2(\boldsymbol{O_0} - \boldsymbol{f})^2}}{\left((\boldsymbol{O_0} - \boldsymbol{f})^2 - \left(\frac{\boldsymbol{L_o}}{2}\right)^2\right)}$$

$$\boldsymbol{L_I} = \frac{\boldsymbol{f L_o}\sqrt{\boldsymbol{f}^2 + (\tan \boldsymbol{\theta_o})^2(\boldsymbol{O_0} - \boldsymbol{f})^2}}{\left((\boldsymbol{O_0} - \boldsymbol{f})^2 - \left(\frac{\boldsymbol{L_o}}{2}\right)^2\right)}$$

Equation S3 *Length of the image plane as a function of L_o, θ_o, and O_0. This defines the size of the detector (or translation range if using a single point detector like a fiber) needed to measure signals across a volume of length L_o with a laser that intersects the optical axis at O_0 and at an angle of θ_o. L_I is the distance between P'_{near} and P'_{far}.*

Derivations for $\tan \boldsymbol{\theta}_{\boldsymbol{i}}$ and $\boldsymbol{L}_{\boldsymbol{I}}$ are given below:

Let: $X = (O_0 - f)$ and **Substitute** $o_n = \left(O_0 - \frac{L_O}{2}\right),\ o_f = \left(O_0 + \frac{L_O}{2}\right)$ into exp. for $\boldsymbol{P'}_{\boldsymbol{near/far}}$

Thus: $i_n = f\frac{o_n}{\left(O_0 - f - \frac{L_O}{2}\right)} = f\frac{o_n}{\left(X - \frac{L_O}{2}\right)},\ i_f = f\frac{o_f}{\left(X + \frac{L_O}{2}\right)},\ \ y'_n = f\frac{-\frac{D_O}{2}}{\left(X - \frac{L_O}{2}\right)},\ \ y'_f = f\frac{\frac{D_O}{2}}{\left(X + \frac{L_O}{2}\right)}$

For $\tan\theta_i = \frac{i_n - i_f}{y'_n - y'_f}$ and $L_I = \sqrt{\left(i_n - i_f\right)^2 + \left(y'_n - y'_f\right)^2}$, **find** $(\boldsymbol{i_n} - \boldsymbol{i_f})$ and $(\boldsymbol{y'_n} - \boldsymbol{y'_f})$

$$(\boldsymbol{i_n} - \boldsymbol{i_f}) = \frac{f o_n}{\left(X - \frac{L_o}{2}\right)} - \frac{f o_f}{\left(X + \frac{L_o}{2}\right)} = f\frac{X\left(o_n - o_f\right) + \frac{L_o}{2}\left(o_n + o_f\right)}{\left(X^2 - \left(\frac{L_o}{2}\right)^2\right)}$$

$$\left(\boldsymbol{y'_n} - \boldsymbol{y'_f}\right) = \frac{-fD_o}{2\left(X - \frac{L_o}{2}\right)} - \frac{fD_o}{\left(X + \frac{L_o}{2}\right)} = \frac{-fD_oX}{\left(X^2 - \left(\frac{L_o}{2}\right)^2\right)}$$

Where: $\left(o_n - o_f\right) = -L_o,\ \left(o_n + o_f\right) = 2O_0,\ X = (O_0 - f)$

Thus $\left(i_n - i_f\right)$ and $\left(y'_n - y'_f\right)$ simplify to:

$$(\boldsymbol{i_n} - \boldsymbol{i_f}) = \frac{L_o f^2}{\left((O_0 - f)^2 - \left(\frac{L_o}{2}\right)^2\right)} = \frac{\boldsymbol{f}}{\left((\boldsymbol{O_0} - \boldsymbol{f})^2 - \left(\frac{\boldsymbol{L_o}}{\boldsymbol{2}}\right)^2\right)} * (\boldsymbol{L_o f})$$

$$\left(\boldsymbol{y'_n} - \boldsymbol{y'_f}\right) = \frac{-fD_o(O_0 - f)}{\left((O_0 - f)^2 - \left(\frac{L_o}{2}\right)^2\right)} = \frac{\boldsymbol{f}}{\left((\boldsymbol{O_0} - \boldsymbol{f})^2 - \left(\frac{\boldsymbol{L_o}}{\boldsymbol{2}}\right)^2\right)} * \left(-\boldsymbol{D_o}(\boldsymbol{O_0} - \boldsymbol{f})\right)$$

Which yields:

$$\tan \boldsymbol{\theta}_{\boldsymbol{i}} = \frac{i_n - i_f}{y'_n - y'_f} = -\frac{\boldsymbol{L_o}}{\boldsymbol{D_o}}\frac{\boldsymbol{f}}{(\boldsymbol{O_0} - \boldsymbol{f})}$$

$$\boldsymbol{L_I} = \sqrt{\left(i_n - i_f\right)^2 + \left(y'_n - y'_f\right)^2} = \frac{\boldsymbol{f}\sqrt{(\boldsymbol{L_o f})^2 + \boldsymbol{D_o}^2(\boldsymbol{O_0} - \boldsymbol{f})^2}}{\left((\boldsymbol{O_0} - \boldsymbol{f})^2 - \left(\frac{\boldsymbol{L_o}}{\boldsymbol{2}}\right)^2\right)}$$

Section 2: Mapping of points from the object plane to the image plane and the system spatial resolution as a function of the collection fiber core diameter and distance.

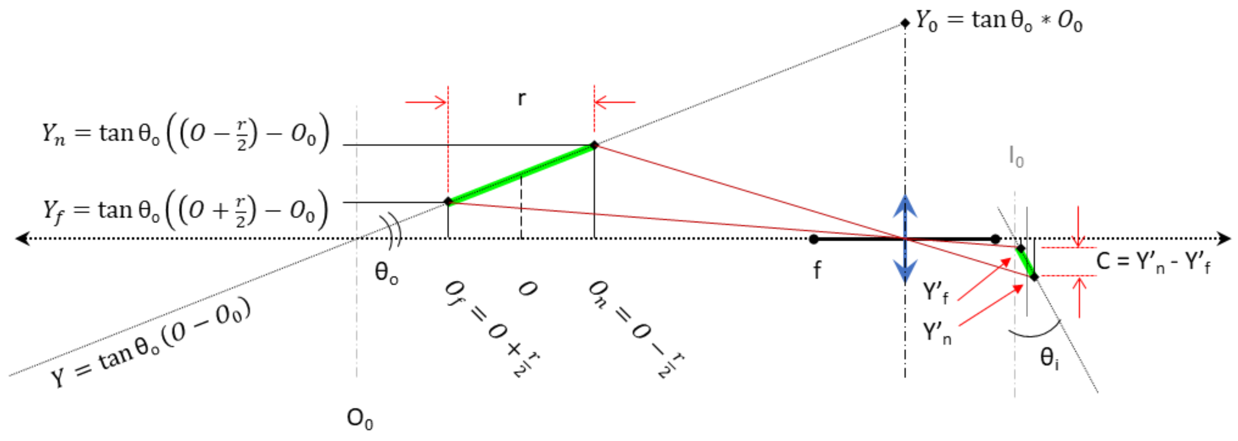

Figure S2 *Imaging a segment of the S-LIDAR excitation laser of length 'r' centered at a distance 'O' from the focusing element (blue double arrow) onto a collection fiber with a core diameter of 'C'. Note that for the system described in this work, θ_o = 2.86°, thus the small angle approximation is used to equate the length of the laser segment with its projection along the 'o' axis.*

Note: figure dimensions exaggerated for clarity.

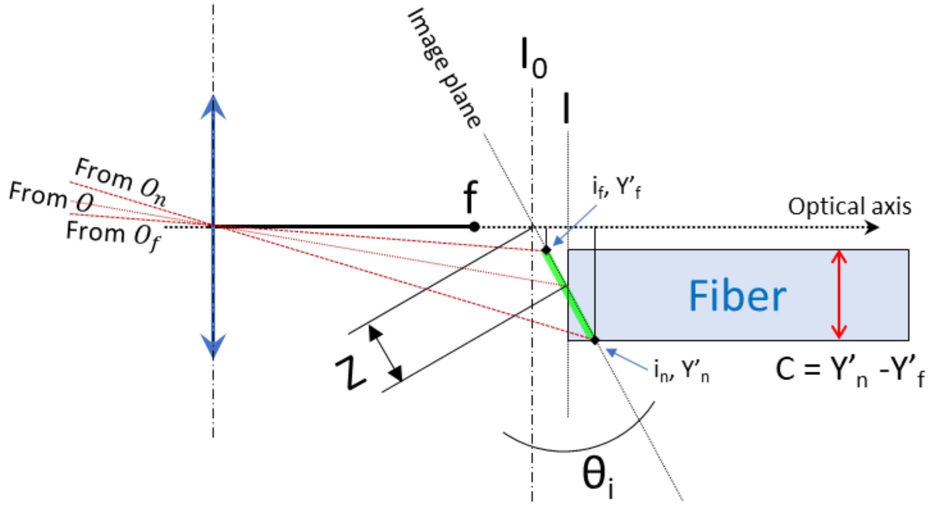

Figure S3 *Image plane detail. Rays originating from points defined by O_f and O_n (see Figure S2 above) arrive at the image plane at points P'$_f$ = (i_f, Y'$_f$) and P'$_n$ = (i_n, Y'$_n$) and fall onto a fiber of diameter 'C', while point O (the center of the measurement) maps to a distance 'Z' along the image plane (measured from I_0).*

Note: figure dimensions exaggerated for clarity.

For an S-LIDAR system (**Figure S2**) with an excitation laser intersecting the optical axis at 'O_0', at an angle of 'θ_o' with respect to the optical axis, and with a signal collection fiber with core diameter 'C' (or detector pixel size), each distance, 'O', along the 'o' axis maps to a point along image plane a distance 'Z' from the intersection with the optical axis. Also, not that each point 'O' is measured with a spatial resolution of 'r' (between O ± $^r/_2$):

Signal Collection Parameters:

$$Z = \frac{\pm f}{(O-f)} \sqrt{\left(O - \frac{O_0(O-f)}{(O_0-f)}\right)^2 + (O-O_0)^2(\tan\theta_o)^2}$$

Where **Z > 0 for O > O_0** and **Z < 0 for O < O_0**

Equation S4 *Position along the image plane, 'Z', which corresponds to the point 'O' (see **Figure S2 and S3**).*

$$r \approx -\frac{C}{f\tan\theta_o}\frac{(O-f)^2}{(O_0-f)}$$

Equation S5 *Spatial resolution for measurement centered at point 'O', that is, the region of length 'r' centered around 'O' (i.e., between O+$^r/_2$ and O-$^r/_2$) from which a signal is collected (see **Figure S2**). See derivation for an exact expression.*

Where f = telescope focal length and O = distance to the measurement point along the optical axis from the focusing element (telescope's mirror/lens). Note that 'r' is the region around the point 'O' for which the collection fiber (core diameter 'C') is collecting a signal.

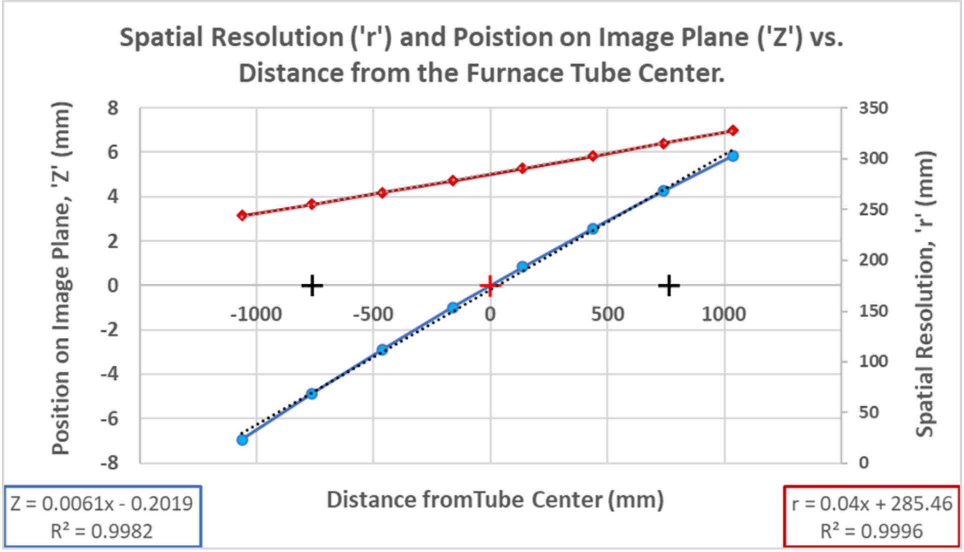

Figure S4 *Plot of 'Z' (Blue) and 'r' (Red) versus position along the furnace tube relative to its center (specifically, O-O_0). The tube end and center points are marked with '+' (ends in Black and center in Red). While 'Z' and 'r' are not linear functions of 'O', fits are provided to demonstrate the fact that these values are approximately linear across the length of the furnace tube.*

Derivation for $'\boldsymbol{Z}'$ and $'\boldsymbol{r}'$ is given below:

For the previously described S-LIDAR system, the laser travels in the '+o' direction (see **Figure S2**) along a line given by:

$$\mathrm{Y} = \tan\theta_o\,(O - O_0), \qquad \tan\theta_o = -\frac{D_o}{L_o}$$

Where D_o and L_o are the dimensions of the furnace tube described in **Section 1**.

Each point along the laser beam, $P = \big(O, \tan\theta_o\,(O - O_0)\big)$, maps to a corresponding point along the image plane:

$$P' = \left(\frac{Of}{(O-f)}, \tan\theta_o\,(O - O_0)\frac{-f}{(O-f)}\right)$$

Shifting the origin of P' from the center of the focusing element to the point $(I_0, 0)$ yields a **vector, 'V',** pointing along the image plane:

$$V = P' - (I_0, 0) = \left(\frac{Of}{(O-f)} - \frac{O_0 f}{(O_0 - f)}, \tan\theta_o\,(O - O_0)\frac{-f}{(O-f)}\right)$$

$$V = P' - (I_0, 0) = \frac{f}{(O-f)}\left(O - \frac{O_0(O-f)}{(O_0-f)}, -(O - O_0)\tan\theta_o\right)$$

Projecting this vector onto the image plane yields (see **Figure S3**):

$$\boldsymbol{Z} = \pm|\boldsymbol{V}| = \frac{\pm\boldsymbol{f}}{(\boldsymbol{O}-\boldsymbol{f})}\sqrt{\left(\boldsymbol{O} - \frac{\boldsymbol{O}_0(\boldsymbol{O}-\boldsymbol{f})}{(\boldsymbol{O}_0-\boldsymbol{f})}\right)^2 + (\boldsymbol{O}-\boldsymbol{O}_0)^2(\tan\boldsymbol{\theta}_o)^2}$$

Where $Z > 0$ for $O > O_0$ and $Z < 0$ for $O < O_0$.

For a fiber with core diameter 'C', its upper and lower edges can be represented as points along the image plane as:

$$C = Y'_n - Y'_f$$

Where only the 'Y' projection is used due to the small size and orientation (i.e., the fiber face perpendicular to the 'i' axis, see **Figure S3**) of the fiber core.

Y'_n and Y'_f can be mapped to points along the laser, Y_n and Y_f, using the lens equation and system magnification:

$$C = Y_n * M_n - Y_f * M_f$$

$$O'_{f,n} = \frac{O_{f,n} f}{(O_{f,n} - f)}, \qquad M_{f,n} = \frac{-f}{(O_{f,n} - f)}$$

Where $\boldsymbol{O_{f,n}} = \ \boldsymbol{O} \pm \frac{r}{2}$ defines the region around the point 'O' from which the system is collecting a signal (see **Figure S2 and S3**). These points, $P_{f,n}$ and $P'_{f,n}$ are given by:

$$P_{f,n} = (O_{f,n}, Y_{f,n}) = \ \left(O \pm \frac{r}{2}, \tan\theta_o \left(O \pm \frac{r}{2} - O_0\right)\right)$$

$$P'_{f,n} = (O'_{f,n}, Y'_{f,n}) = \left(\frac{(O \pm \frac{r}{2})f}{\left((O \pm \frac{r}{2}) - f\right)}, -\tan\theta_o \frac{\left((O \pm \frac{r}{2}) - O_0\right)f}{\left((O \pm \frac{r}{2}) - f\right)}\right)$$

The expression for the fiber core diameter, $\boldsymbol{C} = \ \boldsymbol{Y'_n} - \boldsymbol{Y'_f}$, becomes:

$$C = -f * \tan\theta_o * \left[\frac{(O - \frac{r}{2} - \ O_0)}{(O - \frac{r}{2} - f)} - \frac{(\ O + \frac{r}{2} - O_0)}{(O + \frac{r}{2} - f)}\right]$$

Multiply both sides by $\left(O - \frac{r}{2} - f\right) * \left(O + \frac{r}{2} - f\right)$, let $\mathbf{G} = \boldsymbol{\tan\theta_o \frac{(O_0 - f)}{C}}$, and simplify:

$$(O - f)^2 - \frac{r^2}{4} = f * \left(\tan\theta_o * \frac{(O_0 - f)}{C}\right) * r$$

$$\frac{r^2}{4} + fGr - (O - f)^2 = 0$$

Solving for 'r' yields:

$$r = 2 * \left(-fG \pm \sqrt{(fG)^2 + (O - f)^2}\right)$$

$$\boldsymbol{r = 2fG\left(-1 + \sqrt{1 + \left(\frac{(O - f)}{(fG)}\right)^2}\right)}$$

For $\boldsymbol{\left(\frac{(O-f)}{(fG)}\right)^2 \ll 1}$**,** 'r' becomes:

$$\boldsymbol{r \approx \frac{(O - f)^2}{(fG)}}$$

$$\boldsymbol{r \approx \frac{C}{f\tan\theta_o} \frac{(O - f)^2}{(O_0 - f)}}$$

The exact expression is:

$$r = 2f\tan\theta_o \frac{(O_0 - f)}{C}\left(-1 + \sqrt{1 + \left(\frac{(O - f)}{\left(f\tan\theta_o \frac{(O_0 - f)}{C}\right)}\right)^2}\right)$$
